# Quantifying and Trending the Thermal Signal as an Index of Perfusion in Patients Sedated with Propofol

**DOI:** 10.3390/healthcare6030087

**Published:** 2018-07-24

**Authors:** Surender Rajasekaran, Mark Pressler, Jessica L. Parker, Alex Scales, Nicholas J. Andersen, Anthony Olivero, John R. Ballard, Robert McGough

**Affiliations:** 1 Department of Pediatric Critical Care Medicine, Helen DeVos Children’s Hospital, 100 Michigan Street NE, Grand Rapids, MI 49503, USA; Anthony.Olivero@helendevoschildrens.org; 2 Department of Pediatrics, Michigan State University College of Human Medicine, 15 Michigan Street NE, Grand Rapids, MI 49503, USA; pressler92.mark@gmail.com; 3 Office of Research Administration, Spectrum Health, 100 Michigan Street NE, Grand Rapids, MI 49503, USA; Jessica.Parker2@spectrumhealth.org (J.L.P.); Nicholas.Andersen@spectrumhealth.org (N.J.A.); 4 Department of Emergency Medicine, Helen DeVos Children’s Hospital, 100 Michigan Street NE, Grand Rapids, MI 49503, USA; scalesalex0@gmail.com; 5 Design Solutions, Inc., 1266 Park Road, Chanhassen, MN 55317, USA; john.ballard@design-solutions.com; 6 Department of Electrical and Computer and Engineering, Michigan State University, 2120 Engineering Building, East Lansing, MI 48824, USA; mcgough@egr.msu.edu

**Keywords:** hemodynamics, monitored anesthesia care, perfusion, propofol, thermal imaging

## Abstract

We examined the feasibility of a thermal imager smart phone attachment as a potential proxy of skin perfusion by assessing shifts in skin temperature following administration of the vasodilatory anesthetic propofol. Four limb distal extremity thermal images were taken before propofol administration and at 5-min intervals thereafter during monitored anesthesia. The study enrolled 60 patients with ages ranging from 1.3 to 18 years (mean 10.7 years old) from April 2016 to January 2017. Five minutes following propofol administration, the median temperature differential (delta temperature) between the core and extremity skin significantly decreased in both upper and lower extremities, 7.9 to 3.6 °C (*p* < 0.0001) and 12.1 to 6.9 °C (*p* < 0.0001), respectively. By 10 min, the median delta temperatures further decreased significantly in the upper (*p* = 0.0068) and lower extremities (*p* = 0.0018). There was a concordant decrease in mean blood pressure (MBP). These trends reverted back when the subject awoke. There was no significant difference between the four operators who used the camera (*p* = 0.0831). Blood pressure and time temperature change was the only value of significance. Mobil thermal imaging represents a non-invasive modality to assess perfusion in real time. Further studies are required to validate the clinical utility.

## 1. Introduction

Monitored anesthesia care (MAC) with propofol requires intensive monitoring, as its hemodynamic effects may significantly alter the patient’s cardiovascular state [1]. In clinical practice, it is essential to promptly identify cardiovascular decompensation early so as to initiate appropriate interventions. Presently, serial blood pressure and heart rate monitoring are the gold standards in routine anesthetic monitoring, but vasodilation and vasoplegia may precede changes in blood pressure and heart rate [2]. Health care professionals also use physical exam findings such as distal extremity capillary refill and arterial pulse palpation. These procedures are subjective and often inaccurate. Changes in vasomotor tone caused by propofol such as vasodilation also lead to increased cutaneous heat exchange between the skin and the immediate environment [3].

Infrared imager devices are already being used to measure radiant biological signals in a variety of settings and could serve as an innovation in the hemodynamic monitoring of pediatric patients [4]. The thermal imager in this study is an attachment to standard cell phones that converts the infrared radiation emitted from the patient into digital data to generate a temperature map through an infrared focal plane array device [5]. This allows users to quantify and trend radiant heat loss over time.

Distal extremity vasodilation is a hallmark of propofol sedation. Propofol is a potent vasodilator that increases vessel capacitance and decreases resistance by inhibiting sympathetic tone [2]. We posit that propofol-mediated vasodilation would lead to immediate changes in skin temperature quantifiable by thermal imaging. The goal of the present study was to assess the ability of the thermal imager to trend heat loss that registers as skin temperature from propofol-mediated vasodilation and study reversibility as propofol levels drop and vascular tone returns. This study will serve as a proof of concept of a handheld device to monitor changes in skin temperature as a surrogate to perfusion.

## 2. Materials and Methods

### 2.1. Study Design

This is a prospective observational study without any interventions. We enrolled 65 patients <18 years undergoing esophagogastroduodenoscopy (EGD) in our pediatric sedation unit from April 2016 to January 2017. All subjects underwent propofol-induced sedation. Five subjects were excluded from the analysis secondary due to missing data points. The study was performed with respect to the Declaration of Helsinki. The study and consent were reviewed and approved by the Spectrum Health Institutional Review Board.

### 2.2. Subjects

The study included pediatric patients who were undergoing propofol sedations for EGDs. Patients were excluded from the study if they had a pre-existing cardiovascular co-morbidity, gastro-intestinal bleeding, if drugs other than propofol were used in the sedation, or if the patient was scheduled to undergo an additional procedure.

Pediatric intensivists at Helen DeVos Children’s Hospital (HDVCH) provide MAC through a dedicated pediatric sedation program. Loading doses of 2–2.5 mg of propofol were administered by the physician, and afterwards smaller doses were titrated to maintain monitored anesthesia. Oxygen via nasal cannula is administered to all patients undergoing MAC. Additional interventions, dosages, and procedure times were extracted from the sedation record. The sedation nurse assessed the quality of sedation and recovery using the modified Aldrete sedation scale [6,7,8].

### 2.3. Thermal Imaging

**Figure 1:**
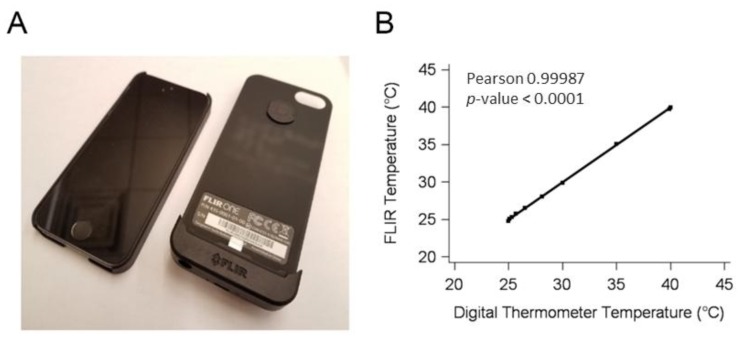
(**A**) The mobile thermal imager (FLIR ONE camera) on the right, and the compatible smartphone on the left (owner name and contact information was blurred from the image). The mobile thermal imager attaches to the back of the smartphone to create a single cohesive device. (**B**) Comparison between the infrared readings of a water bath taken by the mobile thermal imager and a digital thermometer.

The FLIR One thermal imaging camera attachment is a modular attachment to any commercially available smart phone (Figure 1A). The thermal camera used was a FLIR ONE First Generation (s/n FEB5000AAN) (FLIR Systems, Wilsonville, OR, USA), a portable version that attaches to an iPhone 5/5s (Apple Inc., Cupertino, CA, USA). The temperature sensitivity is 0.1 °C with a range of 0–100 °C. We used the FLIR Research IR data acquisition system, which provides the digital tools for image creation and temperature measurement.

Validation of the camera was done using a VWR Sheldon 1226 water bath (VWR International, Radnor, PA, USA) set to an assigned temperature. The temperature was verified with a Thermalert TH5 digital thermometer (Physitemp Instruments Inc., Clifton, NJ, USA). Water temperature was increased in increments of 0.1 °C, 0.2 °C, 0.4 °C, 0.8 °C, 1.6 °C; at 30 °C, temperature was increased by 5 °C to 40 °C. After the TH5 measured the temperature, an image was taken with the FLIR One camera. The investigator waited one minute between equivalent temperature readings, for a total of two images per temperature. The water bath validation results demonstrated a strong linear correlation between camera readings and a digital thermometer (*r* = 0.99) (Figure 1B).

All patients who underwent sedation for an EGD had an oral temperature taken by an electronic thermometer before and after the procedure. Those values were not significantly different (data not shown). The patients were then positioned on their left lateral side prior to sedation with the similarly trained personnel (intensivist, sedation registered nurse, sedation tech) situated in a consistent formation near the bedside, thus allowing for the camera placement and angle to be consistently maintained throughout the procedure. Prior to, and serially during the procedure, a single operator obtained thermal imaging pictures of the soles of the feet and palms of the hand, including all digits using a folded sheet of A4 paper lengthwise (28 cm) to maintain a constant distance. The images were later associated with blood pressure readings taken by the non-invasive oscillometric technique every three minutes. Vital signs and patient demographic data were abstracted from the patient’s record. An ambient room temperature of approximately 21 °C was maintained in each procedure suite. Thermal values were compared with the blood pressure values taken temporally closest to the time of image acquisition. Thermal imaging is not approved by the United States Food and Drug Administration for the purpose of hemodynamic monitoring. In this manuscript, the use of thermal imaging was entirely investigational and not used to change therapy or modify care during MAC.

### 2.4. Data Analysis

**Figure 2:**
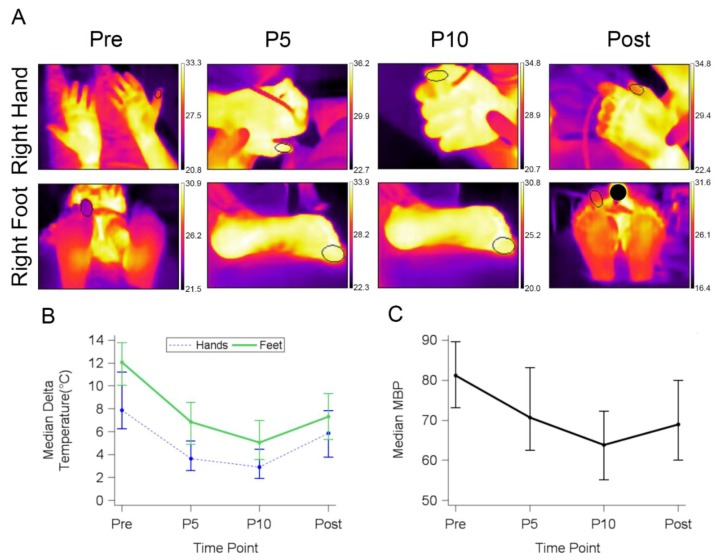
Median delta temperature decreases during propofol sedation. (**A**) Representative serial images before propofol (Pre), at 5 (P5) and 10 (P10) min post-propofol administration, and as the patient awakes (Post). Each row represents one patient over time. Region of interest (ROI; oval) marked during through the pre- and post-sedation time periods. Each image has the corresponding temperature legend in °C. (**B**) Median delta temperature (difference between average ROI reading and initial core) at each time point (Pre, P5, P10, Post). (**C**) Median of all patients’ mean blood pressure (MBP) during propofol sedation and as the patient awakes.

Distal extremity thermal imaging data was collected using infrared software as part of the FLIR One thermal imaging camera attachment package. An ellipse was drawn to fit around the entire pad of the big toe and thumb using the digital photo as a guide to define the region of interest (ROI). The captured pixels each represent an average temperature value, which then generates the average for the entire elliptical region (Figure 2A). Measurement ranges were gathered from each extremity individually and over time. The right and left thumb were averaged and subtracted from the initial core temperature to calculate the delta (core minus distal extremity) temperature for each time point. Four time points were used for collecting thermal images: baseline just prior to propofol administration (pre), five minutes post-propofol administration (P5), 10 min post-propofol administration (P10), and the last image was obtained immediately after when the patient awoke and had purposeful movement (post). Blood pressure readings were collected every three minutes and were compared to the thermal readings.

### 2.5. Statistical Analysis

Descriptive statistics were used to summarize various patient characteristics and outcome measures. Continuous variables were expressed as mean ± standard deviation for normally distributed data and median interquartile range (IQR) for non-normally distributed data. Categorical variables were expressed as frequency (percent). *p*-values for determining the difference between core temperature and mobile thermal imager generated skin temperature were produced from a Friedman’s analysis, since the data was not normally distributed across the four time points. Mixed models examine the relationship between a fixed variable and a variable that represents a random selection from the population. A mixed model was utilized to investigate differences among the four investigators over time. The interaction between the two was not included in the final model because it was not significant. A multivariate regression model was utilized to identify contributing variables that were predictive of delta temperature. To test the validation results, a Pearson correlation coefficient was used. To determine if various variables were correlated with each other, a Spearman correlation coefficient was used, since the data was not normally distributed. All statistical analyses were generated using SAS (SAS Enterprise Guide software, Version 7.1, SAS Institute Inc., Cary, NC, USA).

## 3. Results

### 3.1. Demographics

There were a total of 60 pediatric study participants. Study subject ages ranged from 1.3 to 18 years (mean 10.7 years old). Gender was split with 31 boys and 29 girls. Mean body surface area was 1.3 m^2^ with a 0.4 standard deviation (Table 1). This study was performed on sedated subjects undergoing a scheduled EGD procedure. Patients had no known co-morbidities and were healthy aside from the gastrointestinal troubles that required an endoscope. Total propofol dose ranged from 2.1 to 26.7 mg/kg with a mean of 5.8 mg/kg (Table 1).

### 3.2. Median Peripheral Temperature Changes during Propofol Administration

We normalized surface temperature by subtracting the thermal readings from the initial core temperature at each time point (delta temperature). A normalized delta temperature allowed us to compare temperature changes at each time point across study subjects. To establish a delta temperature baseline (pre), investigators took thermal images of each participant’s hands and feet while lying down prior to propofol administration (Figure 2A). Average temperature within the region of interest was subtracted from the patient’s core temperature. All subjects had similar total (hand and foot) median pre-delta temperature (10.2 °C) (Figure 2B).After propofol induction, the surface hand temperature rose. By five minutes post-propofol (P5), the hand delta temperature decreased significantly from 7.9 °C to 3.6 °C (54.4%) as surface temperature increased. The median hand delta temperature further significantly decreased 19.4% to 2.9 °C by 10 min post-propofol (P10). The temperature change was reversible. The post-median delta temperature rose significantly to 5.9 °C 5 min after the patients awoke from sedation (post) (Figure 2). At post, patients failed to fully recover to the pre-delta temperature; skin retained elevated thermal emission (Figure 2B).

Lower extremity temperatures showed the same trend, but the normalized pre-median delta difference was 4.2 °C higher than the upper extremities (Figure 2B). This is because the pre feet temperatures were cooler than the hands. By P5, feet median delta temperature significantly decreased by 42.7% to 6.9 °C. By P10, median feet temperature significantly decreased another 26.0% to 5.1 °C. As seen in the upper extremities, the post-median feet delta temperature significantly increased but did not reach pre-level (Figure 2).

Compared with the extremity median delta temperature, there was a concordant drop and rise in the subjects’ mean blood pressure (MBP). Prior to propofol the MBP was 81.2 mm/Hg; MBP gradually decreased by 10 mm/Hg by both P5 and P10. By post, blood pressure significantly increased compared with P10, even though the mean blood pressure never reached pre-level prior to discharge (Figure 2C). Correlation analysis did not reveal any correlation between delta temperature and MBP at any time point such as the lowest MBP (P10) *r* = 0.24, *p* = 0.0636. Mean propofol dose (mg/kg) did not correlate with pre (*r* = − 0.17, *p* = 0.20), P5 (*r* = − 0.12, *p* = 0.34), P10 (*r* = − 0.13, *p* = 0.34), or post-delta temperature (*r* = 0.02, *p* = 0.8869).

### 3.3. Multivariate Regression

We performed a multivariate regression to identify contributing variables. The model included the four time points, propofol dose, gender, age, and body surface area (BSA). The only significant predictor was the time point variable (Table 2).

### 3.4. Investigator Differences

**Figure 3:**
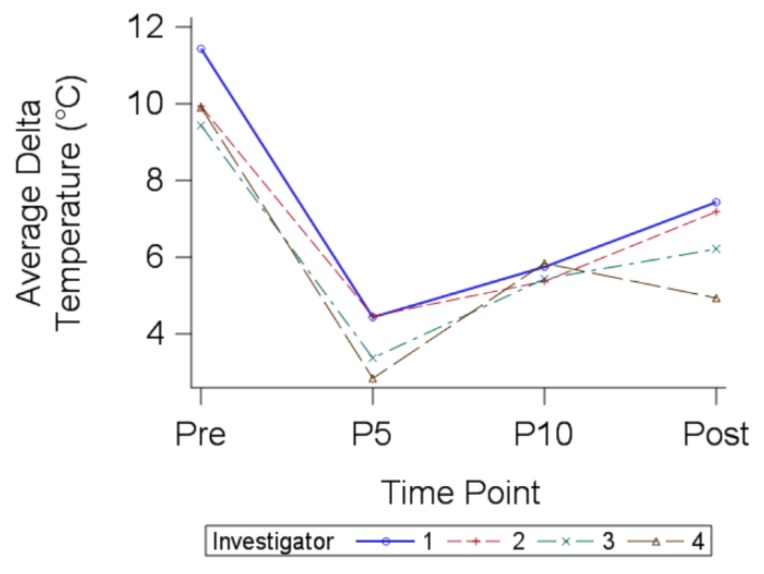
Longitudinal median delta temperatures for each investigator. No significant differences were found between investigators taking thermal readings. Investigator 1 (*n* = 24), Investigator 2 (*n* = 25), Investigator 3 (*n* = 5), Investigator 4 (*n* = 6).

To investigate if there was a difference between the four investigators over time, a mixed model was created including time and investigator. The repeated measuring of variables at fixed time points plus a random sample of different investigators made a mixed model the most appropriate statistical analysis. We found that the investigator and time interaction in the model was not significant, so it was removed (*p* = 0.2471). Investigator intra-study variation was insignificant between all four investigators for all time points, although Investigator 1’s pre-median delta temperature was higher than the other investigators (Figure 3). The P5, P10, and post-median delta temperature were all similar between investigators. We also found that the specific investigator did not matter. There were no significant differences in the variability between investigators (*p* = 0.0831).

## 4. Discussion

In this study, distal extremity temperature rapidly increased longitudinally during propofol sedation. Infusion of propofol preceded a drop in the median delta temperature as the skin warmed and reduced the difference between skin and initial core temperatures. Discontinuation of propofol reversed this trend; delta temperature increased as mean blood pressure trended to normal. Temperature was the only variable that significantly changed after controlling for gender, body size, and propofol dose.

This study provides the possibility of real-time monitoring of skin temperature and that such technique may act as a surrogate for perfusion and pattern recognition during anesthesia. Propofol increases tissue perfusion by inhibiting the sympathetic vasoconstrictive nerve cells resulting in vessel dilation and thermal radiation [2,3,9]. Volatile anesthetic gases exert their effect through similar mechanisms and, as such, their vasodilatory effects could be similarly trended [10]. We found a linear increase in surface temperature that was concordant with a decrease in blood pressure. As propofol’s effect on sedation is relieved, surface temperature begins to fall and mean blood pressure rises. That reversal further substantiates the observation that these effects are due to propofol’s effect on vasculature. Other investigators have shown the concept of skin to core temperature differential to be useful in monitoring circulatory changes [9,11,12,13]. In patients with vascular disease, extremity skin temperature positively correlated with tissue perfusion [13,14]. Researchers monitored thermal radiation to evaluate extremity perfusion in vascular diseases using smart phone attachments [13]. Continuous thermal imagery offers the potential to monitor the real-time therapeutic response to vasoactive agents to improve peripheral tissue perfusion or to immediately discontinue ineffective modalities in other clinical settings [13,14]. As such, the trend the device detects may be more important than the absolute temperature.

Propofol elimination does not follow a one compartment pharmacokinetic model. Propofol disperses into a deep compartment, thereby extending the terminal elimination half-life to greater than 100 minutes [15]. It is still debated whether propofol clearance adheres to a two or three compartment pharmacokinetics [15,16,17,18]. Nevertheless, propofol is detectable hours after administration. Propofol pharmacokinetics may explain that neither the temperature nor the mean blood pressure returned to pre-study levels even though the patient was awake.

This study is distinct from others in that we used a handheld thermal imaging device to map skin surface radiation under anesthesia rather than a thermistor probe that measures only the temperature at the point of contact. This technology allows the capture of a wide thermal emission field and converts it to a single image with several thousand temperature points, recorded in a fraction of a second. Continuous thermal imagery offers the potential to monitor the real-time therapeutic response of vasodilators to improve peripheral tissue perfusion or to immediately discontinue ineffective modalities [19]. We found the device to be reproducible. The device also requires little training, reducing the administrative burden; we recruited four medical students to take the thermal images. Furthermore, the cordless nature of the device does not impede provider access to the patient, as the thermal camera does not require direct patient contact.

The study does have limitations in that the patients were cared for in a setting where ambient temperature was not tightly controlled. Furthermore, not all the patients had a similar baseline blood pressure and heart rate. The patient’s anxiety, discomfort, and stress of being in unfamiliar settings such as sedation suites differentially affect these. However, in studies such as this that measure hemodynamic changes, the patients often serve as their own controls. A further limitation is the variation of propofol dosing prevented a true dose response analysis. Physicians determined the appropriate propofol dose on a case-by-case basis depending on an individual patient’s response and reception of the procedure. Because of this limitation, we were unable to correlate either propofol dose or blood pressure with changes in peripheral temperature.

## 5. Conclusions

This study reveals the feasibility of using a mobile thermal imager to assess temperature changes in patients receiving intravenous propofol. We showed that the measured skin temperature from a mobile thermal imager may serve as a potential surrogate for perfusion. The thermal bio-signal showed promise for use as a novel mode of pattern recognition during anesthesia and hemodynamic monitoring in sedated children, but further verification and validation is required.

## Figures and Tables

**Table 1:** Demographics of the subject population.

Variable	All *n* = 60
Age (years) *	10.7 ± 4.6
Sex, *n* (%)	
Male	31 (51.7)
Female	29 (48.3)
Weight (kg)	44.3 ± 20.2
BSA (m^2^)	1.3 ± 0.4
Sedation length (min)	12.9 ± 3.9
Propofol dose (mg/kg)	5.8 ± 3.4

Analyses are the corresponding mean ± standard deviation. * 49 subjects. BSA: body surface area.

**Table 2:** Regression model predicting differential between core temperature and mobile thermal imager.

Variable	Estimate	*p*-Value
Time (5 min) *	−5.12	<0.0001
Time (10 min) *	−6.23	<0.0001
Time (post) *	−3.36	<0.0001
Propofol dose (mg/kg)	0.07	0.38
Gender (Female) ^#^	0.78	0.14
Age	0.01	0.96
BSA	0.88	0.55

* Pre propofol was the reference point, ^#^ male was the reference gender. BSA: body surface area.
